# Clinical and transcriptomic features of persistent exacerbation‐prone severe asthma in U‐BIOPRED cohort

**DOI:** 10.1002/ctm2.816

**Published:** 2022-04-26

**Authors:** Uruj Hoda, Stelios Pavlidis, Aruna T. Bansal, Kentaro Takahashi, Sile Hu, Francois Ng Kee Kwong, Christos Rossios, Kai Sun, Pankaj Bhavsar, Matthew Loza, Frederic Baribaud, Pascal Chanez, Stephen J. Fowler, Ildiko Horvath, Paolo Montuschi, Florian Singer, Jacek Musial, Barbro Dahlen, Norbert Krug, Thomas Sandstrom, Dominic E. Shaw, Rene Lutter, Louise J. Fleming, Peter H. Howarth, Massimo Caruso, Ana R. Sousa, Julie Corfield, Charles Auffray, Bertrand De Meulder, Diane Lefaudeux, Sven‐Erik Dahlen, Ratko Djukanovic, Peter J. Sterk, Yike Guo, Ian M. Adcock, Kian Fan Chung, the U‐BIOPRED study group

**Affiliations:** ^1^ National Heart and Lung Institute Imperial College London, and Biomedical Research Unit Royal Brompton and Harefield NHS Trust London UK; ^2^ Department of Computing & Data Science Institute Imperial College London; ^3^ Acclarogen Cambridge UK; ^4^ Research Centre for Allergy and Clinical Immunology Asahi General Hospital Asahi Japan; ^5^ Janssen Research and Development High Wycombe Buckinghamshire UK; ^6^ Assistance Publique des Hôpitaux de Marseille, Clinique des Bronches Allergies et Sommeil Aix Marseille Université Marseille France; ^7^ Division of Infection Immunity and Respiratory Medicine Faculty of Biology School of Biological Sciences Medicine and Health University of Manchester, Manchester Academic Health Science Centre, and NIHR Biomedical Research Centre Manchester University Hospitals NHS Foundation Trust Manchester UK; ^8^ Department of Pulmonology Semmelweis University Budapest Hungary; ^9^ Catholic University of Sacred Heart Rome Italy; ^10^ Department of Respiratory Medicine University Children's Hospital Zurich and Childhood Research Center Zurich, and Department of Paediatrics Inselspital University of Bern Switzerland; ^11^ Department of Medicine Jagiellonian University Medical College Krakow Poland; ^12^ Centre for Allergy Research Karolinska Institutet Stockholm Sweden; ^13^ Fraunhofer Institute for Toxicology and Experimental Medicine Hannover Germany; ^14^ Department of Public Health and Clinical Medicine Umeå University Umeå Sweden; ^15^ Respiratory Research Unit University of Nottingham UK; ^16^ Academic Medical Centre University of Amsterdam Amsterdam The Netherlands; ^17^ NIHR Southampton Biomedical Research Centre Clinical and Experimental Sciences Faculty of Medicine University of Southampton UK; ^18^ Department of Biochemical and Biotechnological Medicine University of Catania Catania Italy; ^19^ Respiratory Therapeutic Unit GSK Stockley Park UK; ^20^ AstraZeneca R&D, Molndal Sweden, and Areteva R&D Nottingham UK; ^21^ European Institute for Systems Biology and Medicine CNRS‐ENS‐UCBL‐INSERM Lyon France

**Keywords:** asthma exacerbations, severe asthma, CEACAM5, frequent exacerbators, persistent frequent exacerbators

## Abstract

**Background:**

Exacerbation‐prone asthma is a feature of severe disease. However, the basis for its persistency remains unclear.

**Objectives:**

To determine the clinical and transcriptomic features of frequent exacerbators (FEs) and persistent FEs (PFEs) in the U‐BIOPRED cohort.

**Methods:**

We compared features of FE (≥2 exacerbations in past year) to infrequent exacerbators (IE, <2 exacerbations) and of PFE with repeat ≥2 exacerbations during the following year to persistent IE (PIE). Transcriptomic data in blood, bronchial and nasal epithelial brushings, bronchial biopsies and sputum cells were analysed by gene set variation analysis for 103 gene signatures.

**Results:**

Of 317 patients, 62.4% had FE, of whom 63.6% had PFE, while 37.6% had IE, of whom 61.3% had PIE. Using multivariate analysis, FE was associated with short‐acting beta‐agonist use, sinusitis and daily oral corticosteroid use, while PFE was associated with eczema, short‐acting beta‐agonist use and asthma control index. CEA cell adhesion molecule 5 (*CEACAM5)* was the only differentially expressed transcript in bronchial biopsies between PE and IE. There were no differentially expressed genes in the other four compartments. There were higher expression scores for type 2, T‐helper type‐17 and type 1 pathway signatures together with those associated with viral infections in bronchial biopsies from FE compared to IE, while there were higher expression scores of type 2, type 1 and steroid insensitivity pathway signatures in bronchial biopsies of PFE compared to PIE.

**Conclusion:**

The FE group and its PFE subgroup are associated with poor asthma control while expressing higher type 1 and type 2 activation pathways compared to IE and PIE, respectively.

## Graphical abstract

- In severe asthma, frequent exacerbator (FE) and persistent frequent exacerbator (PFE) are associated with poorer asthma control compared to infrequent exacerbator (IE) and persistent IE, respectively.
- CEACAM5 was the differentially‐expressed transcript in bronchial biopsies in FE compared to IE.
- In FE and persistent FE, there was an increase in the expression of type 1 and type 2 inflammatory pathways.

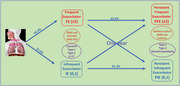

## 1 INTRODUCTION

Asthma exacerbations are episodes of deterioration in symptoms outside of the normal daily variation, associated with airflow obstruction, that require patients to increase their treatment or seek urgent medical attention.^1^ These represent important events in the lifetime of an asthmatic patient because they are associated with a significant degree of morbidity for patients^2^ and have been linked with an accelerated decline in lung function.^3^ Severe asthma is a heterogeneous condition that has been defined ‘as asthma that requires treatment with high dose inhaled corticosteroids plus a second controller and/or systemic corticosteroids to prevent it from becoming “uncontrolled” or that remains “uncontrolled” despite this therapy’.^4^ Up to 54% of patients with severe asthma continue to experience at least two to three exacerbations per year despite optimal treatment.^5^, ^6^ Severe asthma patients with frequent exacerbations have been characterised in women with late‐onset asthma, often obese with near‐normal lung function.^7^, ^8^ Other associated characteristics of these patients include poor asthma control, a lower quality of life, higher sputum eosinophils, a rapid decline in forced expiratory volume in 1 second/force vital capacity (FEV_1_/FVC) ratio, high levels of exhaled nitric oxide and being on higher doses of inhaled and oral corticosteroids (OCS).^9^, ^10^, ^11^

An ATS/EAACI statement on severe asthma exacerbations has emphasised the importance of determining the risk factors and characterising the phenotype or endotype of the frequent exacerbator.^12^ To understand the pathophysiological factors underlying the frequent exacerbations, we compared not only the clinical characteristics but also the proteomic and transcriptomic features of the frequent exacerbator (FE) with those of the infrequent exacerbator (IE) in the European U‐BIOPRED severe asthma cohort.^13^ To further strengthen the analysis, we also compared those FEs who remained FEs over a 1‐year follow‐up period labelled persistent FEs (PFEs) for those who were persistent IEs (PIEs). We analysed the differentially‐expressed genes and the expression of the gene signatures for various inflammatory and immune pathways in blood, sputum and bronchial biopsies and brushings between FE and IE and between PFE and PIE.

## 2 METHODS

### 2.1 Study design

The U‐BIOPRED cohort consisted of current smokers with severe asthma (CSA), ex‐smokers with severe asthma (ESA), nonsmokers with severe asthma (NSA) and nonsmoking healthy volunteers (NH) (Table S1).^6^ At a baseline visit, blood, sputum and urine samples were obtained for the analysis of markers of inflammation and for transcriptomic and proteomic analyses. Differential blood and induced sputum cell counts, serum total immunoglobulin‐E (IgE) and skin prick tests, and fractional exhaled nitric oxide (FeNO) and pre‐ and postbronchodilator spirometry were obtained. Some participants underwent fibreoptic bronchoscopy for bronchial biopsies and bronchial brushings, and nasal brushings were obtained.^14^ The study was approved by the Ethics Committees of each recruiting centre. All subjects gave written and signed informed consent.

At the baseline visit, patients had baseline investigations and clinical data collected. They were asked the number of exacerbations that needed systemic corticosteroid therapy they had experienced in the previous year. Patients were classified as either FE (two or more exacerbations in the past year) or IE (a maximum of one exacerbation in the past year). At a longitudinal visit at 1 year, asthma patients who attended had the baseline investigations repeated, and clinical data were collected, including clinical symptoms and severe exacerbations during the past year. The persistence of the exacerbation phenotype was analysed by classifying those patients who were IE at both visits (persistent IE, PIE) and those who were FE at both visits (persistent FE, PFE).

### 2.2 Transcriptomic and proteomic analyses

RNA from baseline blood samples was isolated using the PAXgene Blood RNA kit (PreAnalytiX, Hombrechtikon, Switzerland) with on‐column DNase treatment (QIAGEN, Valencia, CA). Total RNA was extracted from nasal and bronchial biopsies, epithelial brushings and sputum cells using the miRNeasy mini kit (Qiagen, CA, USA).

Expression profiling of the transcriptome was performed using GeneChip^®^ Human Genome U133 Plus 2.0 Array (Affymetrix, Santa Clara, CA). Pathway analysis, enrichment analysis and functional clustering of differentially expressed genes were performed as described previously^14^, ^15^ ^,^ and protein interaction analysis using annotated protein‐coding genes was performed by STRING version 10.0 (STRING CONSORTIUM 2016, http://www.string‐db.org).^16^ A total of 1129 analytes in serum and sputum supernatants were quantified using SOMAscan v3 (SomaLogic, Boulder, CO; www.somalogic.com) and reported as relative fluorescence units, cross‐plate calibrated and median normalised.

### 2.3 Gene set variation analysis

Gene set variation analysis (GSVA) was used to calculate sample‐wise enrichment scores (ESs)^17^ for 103 predefined gene sets from Molecular Signatures Database v5.2 (MSigDB) (http://software.broadinstitute.org/gsea/msigdb) or from published studies^18^ that relate to airway inflammation and asthma pathogenesis.^19^ GSVA was performed in R using the Bioconductor GSVA package to estimate the variation in gene set enrichment.^17^ One‐way ANOVA was used to analyse the ES differences among group means, and post hoc analysis was performed using Tukey's test. The criteria for a significant change between groups were set at a log_10_ fold‐change of 2.0 and a *p* value <0.05.

### 2.4 Statistical analysis

Clinical variables were summarised as the mean ± standard deviation when continuously symmetrically distributed, as the median and interquartile range when skewed and as their frequencies (proportion) when categorical. Between‐group comparisons were performed with the Mann–Whitney U test or chi‐square tests, as appropriate. Variables with raw *p*<0.05 were modelled in a joint multivariate logistic regression model. Three categories of smoking, namely, nonsmoker, ex‐smoker and current smoker, were coded using indicator variables to avoid any assumptions of a dose effect. Backward step‐wise regression was applied using the Akaike information criterion (AIC).

All clinical as well as transcriptomic and proteomic data sets were uploaded into tranSMART, an open‐source knowledge management platform.^20^ Protein expression data were analysed using multivariate analysis of variance (MANOVA) with age, sex and systemic corticosteroid (SCS) use as covariates. Differentially expressed gene (DEG) data analysis was performed on ArrayStudio® software (OmicSoft®, Cary, NC) applying a general linear model adjusting for age and sex (Bioconductor limma package for R). A Benjamini–Hochberg false discovery rate (FDR) correction was applied. A fold change ≥2.0 and FDR <0.05 were considered nominally statistically significant in transcriptomic and proteomic analyses. Statistical analyses were performed by R version 3.3.1 (R Core Team, 2016).

## 3 RESULTS

### 3.1 Frequent exacerbators and infrequent exacerbators

In total, 420 participants with severe asthma were studied at baseline. FE (*n* = 264; 62.9%) had a greater proportion of female participants (67 vs. 53%; *p* = 0.007) and earlier age of diagnosis (24.8 vs. 29.8 years; *p* = 0.01) than IE (*n* = 156; 37.1%), but there were more current smokers in IE than in FE (Table 1). Sinusitis (38 vs. 26%; *p* = 0.018) and eczema (39 vs. 28%; *p* = 0.042) were more prevalent in the FE group. FE had higher daily short‐acting β‐agonist (SABA) usage (89% vs. 76%; *p* = <0.001) and greater symptom scores (mean ACQ5 2.44 vs. 1.98; *p*<0.001) at baseline, but there was no difference in lung function parameters. Serum IgE was higher in IE (144 vs. 106 IU/ml; *p* = 0.026).

**TABLE 1:** Baseline clinical characteristics of frequent exacerbators (≥2/year) and infrequent exacerbators (<2/year)

	Infrequent	Frequent	*p* Value
N	156	264	
Sex: female^a^	83/156 (53.2)	177/264 (67.1)	0.007
Age (yr)^b^	52.53 (1.06) [156]	51.67 (0.83) [264]	0.449
BMI (kg/m^2^)^b^	28.74 (0.42) [156]	29.53 (0.42) [264]	0.560
Age at diagnosis(year)^b^	29.78 (1.55) [148]	24.8 (1.13) [262]	0.010
Smoking status^a^			
Current smoker	22/156 (14.1)	20/264 (7.6)	0.047
Ex‐smoker	39/156 (25)	76/264 (28.8)	0.467
Never smoked	95/156 (60.9)	168/264 (63.6)	0.648
Atopy test positive^a^	111/133 (83.5)	163/225 (72.4)	0.025
Allergic rhinitis diagnosed^a^	72/140 (51.4)	135/237 (57.0)	0.349
Diabetes diagnosed^a^	13/145 (9.0)	29/250 (11.6)	0.516
Eczema diagnosed^a^	41/146 (28.1)	96/248 (38.7)	0.042
Non‐allergic rhinitis diagnosed^a^	22/143 (15.4)	37/241 (15.4)	1.000
Sinusitis diagnosed^a^	37/144 (25.7)	94/248 (37.9)	0.018
Nasal polyps diagnosed^a^	53/147 (36.1)	84/244 (34.4)	0.828
Osteoporosis diagnosed^a^	31/141 (22.0)	69/246 (28.1)	0.234
GORD diagnosed^a^	74/146 (50.7)	124/241 (51.5)	0.967
Regular xanthine use^a^	23/146 (15.8)	57/242 (23.6)	0.087
Regular leukotriene modifier use^a^	63/149 (42.3)	120/254 (47.2)	0.389
Regular use of LAMA^a^	36/144 (25)	55/236 (23.3)	0.801
Regular SABA use^a^	117/154 (76.0)	224/251 (89.2)	p < 0.001
Regular omalizumab use^a^	24/145 (16.6)	42/239 (17.6)	0.906
Regular OCS use^a^	58/148 (39.2)	123/249 (49.4)	0.061
FEV_1_ (% predicted)^b^	68.36 (1.66) [156]	66.81 (1.34) [261]	0.455
FVC (% predicted)^b^	89.17 (1.52) [156]	86.96 (1.2) [261]	0.166
Residual volume (L)^b^	2.61 (0.07) [122]	2.77 (0.08) [177]	0.612
sGaw (s^−1^ kPa^−1^)^b^	0.93 (0.07) [115]	0.89 (0.06) [173]	0.219
TLC (L)^b^	5.85 (0.04) [156]	5.82 (0.03) [263]	0.550
IgE (IU/ml)^c^	144 (71‐368) [152]	106 (41‐310) [253]	0.026
FeNO (ppb)^c^	27 (15‐52) [146]	24.5 (15‐46) [247]	0.326
Blood eosinophils (%)^c^	2.95 (1‐6) [153]	2.91 (1‐5) [254]	0.354
Blood neutrophils (%)^c^	60.9 (55‐68) [153]	62.98 (56‐71) [254]	0.155
Sputum eosinophils (%)^c^	3.98 (1‐14) [65]	2.9 (0‐16) [115]	0.830
Sputum neutrophils (%)^c^	52.69 (43‐71) [65]	55.1 (32‐75) [115]	0.909
Mean ACQ5^b^	1.98 (0.1) [136]	2.44 (0.08) [236]	p < 0.001
Mean AQLQ^b^	4.67 (0.11) [135]	4.35 (0.08) [232]	0.015
Total HADS^b^	11.5 (0.79) [108]	13.32 (0.6) [186]	0.060
Total SNOT20^b^	31.43 (1.52) [138]	31.97 (1.11) [241]	0.712
Total ESS^b^	7.35 (0.37) [137]	7.76 (0.29) [234]	0.372

Abbreviations: ACQ5, Asthma Control Questionnaire (five questions); AQLQ, asthma quality of life questionnaire; BMI, body metabolic index; ESS, Epworth Sleepiness Scale; FeNO, fractional level of nitric oxide in exhaled breath; FEV1, forced expiratory volume in one second; FVC, forced vital capacity; GORD, gastro‐oesophageal reflux disease; HADS, Hospital Anxiety and Depression Scale; IgE, immunoglobulin E; LAMA, long‐acting muscarinic antagonist; OCS, oral corticosteroid; Ppb, parts per billion; SABA, short‐acting β‐agonist; sGaw, specific airway conductance; SNOT20, sinonasal outcome test (20 questions); TLC, total lung capacity.

^a^ Number (percentage).

^b^ Mean (SD).

^c^ Median (interquartile range).

Those variables with *p*<0.05 in the Mann–Whitney test were modelled in a joint multivariate logistic regression model. To perform backward step‐wise regression, certain variables with high levels of missing data, namely, atopy, residual volume, specific airway conductance, Asthma Control Questionnaire (ACQ) score, Asthma Quality of Life Questionnaire (AQLQ) score, Hospital Anxiety and Depression Scale (score) and sleep score (ESS) score, were removed from consideration. The final model resulted from applying backward step‐wise regression on 17 variables, for which 302 patients had complete data. Variables retained in the model included being an ex‐smoker (compared to nonsmokers and current smokers combined), SABA use, diagnosis of sinusitis and age of onset (all at *p*<0.05) (Table 2A).

**TABLE 2:** Multivariate logistic regression analyses

A.	Joint logistic model of frequent exacerbators at baseline visit (≥2/year)			
Variable	Odds ratio	2.5% CI	97.5% CI	*p* Value
Ex‐smoker	2.32	1.26	4.42	0.008
SABA	2.33	1.16	4.77	0.019
Sinusitis	1.86	1.07	3.30	0.030
Age of onset	0.99	0.97	1.00	0.044
OCS	1.60	0.96	2.70	0.076
FVC (% predicted)	0.99	0.97	1.00	0.083
Male	0.65	0.38	1.10	0.109
Eczema	1.59	0.91	2.83	0.110
Omalizumab	0.59	0.30	1.18	0.131

B.	Joint logistic model of persistent frequent exacerbators (≥2/year)			
	Odds ratio	2.5% CI	97.5% CI	*p* Value
Eczema	4.74	1.77	14.32	0.003
SABA use	4.70	1.96	11.76	0.001
Mean ACQ5	1.66	1.16	2.44	0.007
Total IgE	1.00	1.00	1.00	0.079
Exhaled NO	0.98	0.97	1.00	0.010
Male	0.47	0.20	1.07	0.071
Current smoker	0.20	0.06	0.66	0.009

Abbreviations: ACQ5, Asthma Control Questionnaire (five questions); CI, confidence interval; FVC, forced vital capacity; IgE, immunoglobulin E; NO, nitric oxide; OCS, oral corticosteroids; SABA, short‐acting β agonist.

### 3.2 Persistent frequent exacerbators and persistent infrequent exacerbators

A total of 317 out of 420 severe asthma patients attended a second longitudinal visit for clinical assessment. Of the 198 participants who were FE at the baseline visit, 63.6% remained FE at the longitudinal visit, and of the 119 who were IE at the baseline visit, 61.3% remained IE (Figure S1). There was a greater proportion of females (64% vs. 37%; *p*<0.001) in the PFE group, and the age of onset was earlier (22.8 vs. 29.0 years; *p* = 0.04) than in the PIE group (Table 3). The prevalence of eczema (42.24% vs. 19.12%, *p* = 0.002) and osteoporosis (34.48% vs. 19.4%, *p* = 0.046) was also greater in the PFE group, and they had a greater use of maintenance OCS, SABA and xanthines. PFE were more symptomatic with worse AQLQ and ACQ scores. Levels of serum IgE, exhaled NO and blood eosinophil counts were lower in PFE, but blood neutrophil counts were not significantly different (Table 3).

**TABLE 3:** Clinical characteristics of persistent frequent exacerbators (PFEs) and persistent infrequent exacerbators (PIEs)

	Persistent IE	Persistent FE	*p* Value
N	73	126	
Sex: female^a^	27/73 (36.99)	81/126 (64.29)	*p* < 0.001
Age (year)^b^	53.7 (12.29) [73]	51.85 (12.55) [126]	0.374
BMI (kg/m^2^)^b^	28.44 (4.56) [73]	30.05 (6.72) [126]	0.206
Age at diagnosis(yr)^b^	29.03 (20.13) [70]	22.74 (17.19) [125]	0.042
Smoking status^a^			
Current smoker	15/73 (20.55)	12/126 (9.52)	0.048
Ex‐smoker	16/73 (21.92)	30/126 (23.81)	0.896
Never smoked	42/73 (57.53)	84/126 (66.67)	0.256
Atopy test positive^a^	58/73 (79.45)	83/125 (65.87)	0.110
Allergic rhinitis diagnosed^a^	33/66 (50)	60/113 (53.1)	0.806
Diabetes diagnosed^a^	5/67 (7.46)	16/118 (13.56)	0.310
Eczema diagnosed^a^	13/68 (19.12)	49/116 (42.24)	0.002
Non‐allergic rhinitis diagnosed^a^	10/67 (14.93)	16/114 (14.04)	1.000
Sinusitis diagnosed^a^	19/68 (27.94)	49/117 (41.88)	0.082
Nasal Polyps diagnosed^a^	29/69 (42.03)	40/118 (33.9)	0.340
Osteoporosis diagnosed^a^	13/67 (19.4)	40/116 (34.48)	0.046
GORD diagnosed^a^	29/69 (42.03)	61/115 (53.04)	0.195
Regular xanthine use^a^	11/73 (15.07)	36/125 (28.8)	0.044
Regular leukotriene modifier use^a^	23/73 (31.51)	54/125 (43.2)	0.140
Regular use of LAMA^a^	21/73 (28.77)	34/125 (27.2)	0.942
Regular SABA Use^a^	39/73 (53.42)	105/126 (83.33)	*p* < 0.001
Regular omalizumab use^a^	6/73 (8.22)	12/126 (9.52)	0.958
Regular OCS use^a^	24/73 (32.88)	67/126 (53.17)	0.009
FEV1 (% predicted)^b^	65.61 (19.02) [73]	66.02 (21.3) [124]	0.945
FVC (% predicted)^b^	87.81 (18.2) [73]	86.32 (19.94) [124]	0.532
Residual volume (L)^b^	2.78 (0.76) [56]	2.87 (1.25) [84]	0.677
sGaw (s^−1^ kPa^−1^)^b^	0.95 (0.61) [53]	0.82 (0.75) [84]	0.045
TLC (L)^b^	5.86 (0.51) [73]	5.84 (0.45) [125]	0.871
IgE (IU/ml)^c^	136 (90‐440) [73]	108.5 (42‐264) [122]	0.033
FeNO (ppb)^c^	31 (19‐62) [70]	22.16 (14‐39) [116]	0.035
Blood eosinophils (%)^c^	3.17 (2‐6) [72]	2.58 (1‐5) [121]	0.021
Blood neutrophils (%)^c^	60.38 (55‐67) [72]	62 (56‐71) [121]	0.208
Sputum eosinophils (%)^c^	4.46 (1‐12) [34]	2.7 (0‐9) [55]	0.153
Sputum neutrophils (%)^c^	51.66 (43‐64) [34]	55.1 (35‐75) [55]	0.698
Mean ACQ5^b^	1.8 (1.0) [70]	2.6 (1.22) [124]	*p* < 0.001
Mean AQLQ^b^	4.87 (1.27) [72]	4.19 (1.11) [125]	*p* < 0.001
Total HADS^b^	11.51 (7.6) [72]	13.73 (8.24) [124]	0.065
Total SNOT20^b^	29.55 (17.02) [71]	33.96 (18.48) [124]	0.103

Abbreviations: ACQ5, Asthma Control Questionnaire (five questions); AQLQ, Asthma quality of life questionnaire; BMI, body metabolic index; ESS, Epworth Sleepiness Scale; FeNO, fractional level of nitric oxide in exhaled breath; FEV_1_, forced expiratory volume in 1 second; FVC, forced vital capacity; GORD, gastro‐oesophageal reflux disease; HADS, Hospital Anxiety and Depression Scale; IgE, immunoglobulin E; LAMA, long‐acting muscarinic antagonist; OCS, oral corticosteroid; ppb. parts per billion; SABA, short‐acting β‐agonist; sGaw, specific airway conductance; SNOT20, sinonasal outcome test (20 questions); TLC, total lung capacity.

^a^ Number (percentage).

^b^ Mean (SD).

^c^ Median (Interquartile range).

After backward step‐wise regression, the joint multivariate logistic regression model included SABA use, presence of eczema, current smoking (compared to nonsmokers and ex‐smokers), FeNO and ACQ 5 score (all *p*<0.05). Current smokers had an estimated reduced risk of being a PFE, possibly due to reverse causality (Table 2B).

### 3.3 Differentially expressed genes and proteins

The clinical profile of patients who provided samples for transcriptomic analysis is shown in Table S1. CEA cell adhesion molecule 5 (CEACAM5) was the only significantly differentially‐expressed single gene between FE and IE in the bronchial biopsy samples. No other genes were significantly differentially‐expressed in the other compartments. There was no difference in expression in any compartment between PFE and PIE. Using the Somalogic platform, no proteins were significantly different between IE and FE or between PIE and PFE in serum or sputum supernatant compartments.

### 3.4 GSVA: Comparing FE to IE

**FIGURE 1:**
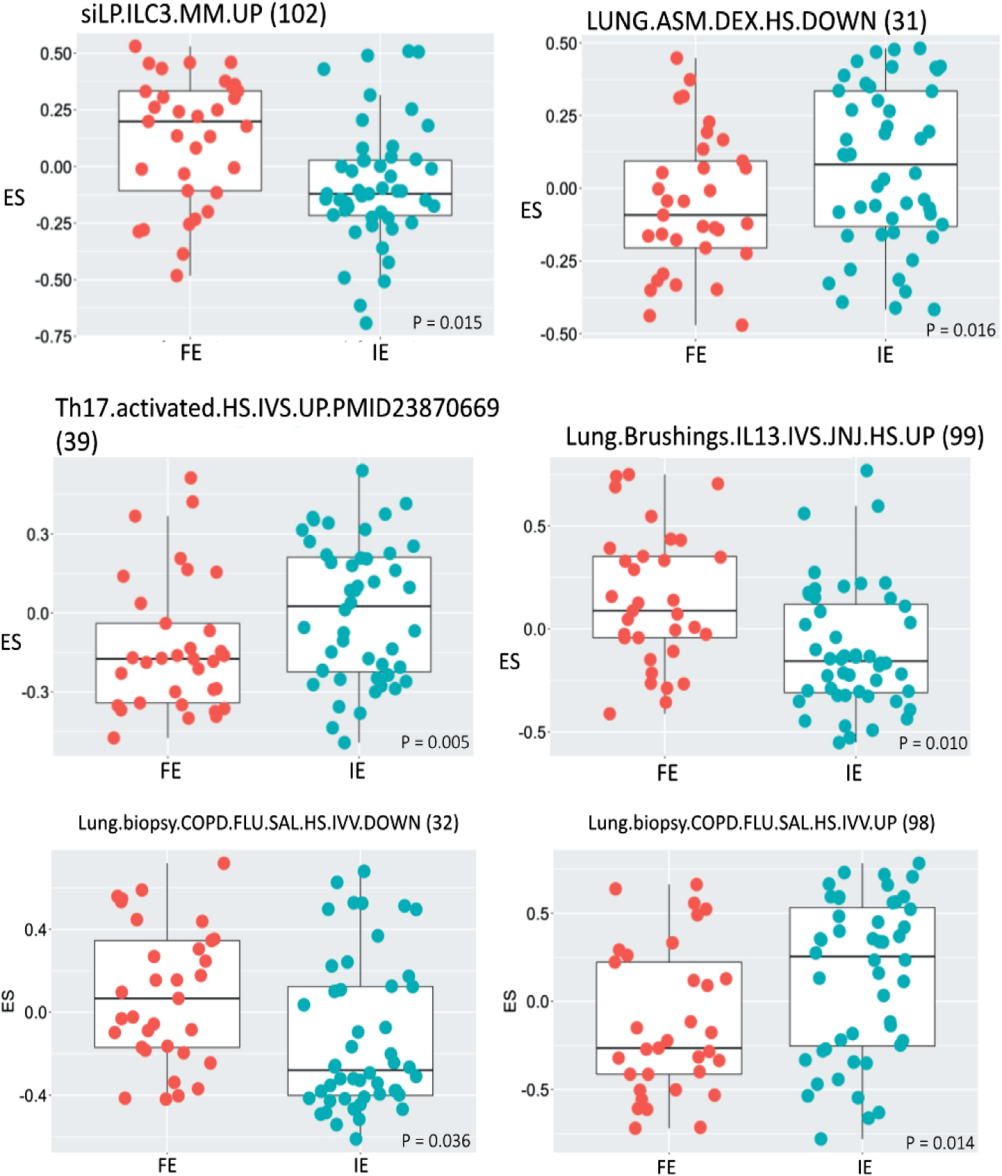
Expression scores (ES) of gene signatures in endobronchial biopsies of frequent exacerbators (FE) compared to infrequent exacerbators (IE). The gene signature number indicates the signature tabulated in Table S2. The *p* significance value is as indicated

Using GSVA, we found that 21 gene signatures out of 103 (nine for nasal brushings; six for bronchial biopsies and seven for sputum cells) were differentially‐enriched in FE compared to IE (Figure 1 and Table 4). No signatures were differentially‐enriched in blood or bronchial brushings.

- *Steroid response‐associated signatures*

**TABLE 4:** Differences in enrichment scores by gene set variation analysis in frequent exacerbators compared to infrequent exacerbators

Pathway	Gene Signature	Signature number	Sputum	Nasal brushings	Bronchial biopsy
Asthma	BAL.eosinophils.WLAC.HS.IVV.UP	53	0.20		
Autoimmune	PBMC.MS.HS.IVV.UP	15		0.17	
Fibrosis	Lung.biopsy.bleomycin.MM.IVV.D35.UP	76	−0.16		
Innate cells	Macrophage.GM_CSF.TNF.PGE2.P3C.HS.IVS.DOWN	94	−0.15		
	Macrophage.GM_CSF.LPSc.HS.IVS.DOWN	90	−0.16		
	Macrophage.GM_CSF.IFNg.HS.IVS.DOWN	86	−0.16		
	Neutrophil.activated.HS.IVS	4		0.24	
	Macrophage.GM_CSF.IFNg.HS.IVS.UP	85		0.19	
	Macrophage.GM_CSF.TNF.PGE2.P3C.HS.IVS.UP	93		0.15	
	Monocyte.activated.HS.IVS	3		0.16	
	Macrophage.GM_CSF.IL4.HS.IVS.DOWN	94		0.21	
	siLP.ILC3.MM.UP	102			0.17
Steroid effects	LUNG.ASM.DEX.HS.DOWN	98			−0.15
	Lung.biopsy.COPD.FLU.SAL.HS.IVV.DOWN	32			0.19
	Lung.biopsy.COPD.FLU.SAL.HS.IVV.UP	31			−0.26
T cells and cytokines	Th17.activated.HS.IVS.UP.PMID23870669	39	−0.16		−0.18
	Lung.ASM.IL17a.healthy_mild.asthma.HS.IVS.UP	47		0.21	
	Lung.Brushings.IL13.IVS.JNJ.HS.UP	99			0.20
Viral inflammation	Lung.biopsy.PolyIC.MM.IVV.72 h.UP	62	−0.18		
	Lung.biopsy.PolyIC.MM.IVV.24 h.UP	58		0.21	
	Lung.biopsy.PolyIC.MM.IVV.48 h.UP	60		0.23	

*Note*: RED indicates higher enrichment score values in frequent exacerbators, and BLUE indicates lower enrichment score values.

Two gene signatures comprising genes whose expression was previously implicated in bronchial biopsies of chronic obstructive pulmonary disease (COPD) patients treated with fluticasone and salmeterol^21^ were enriched in bronchial biopsies of FE compared with IE, with the upregulated genes having a greater ES and the downregulated genes having a lower ES (Table 4 and Table S2, signatures 31 and 32). A gene signature comprising genes downregulated by dexamethasone ex‐vivo in airway smooth muscle cells (Table S2, signature 98) was decreased in bronchial biopsies of FE subjects, suggesting that they have a greater corticosteroid response signature than IE subjects.

(ii) *Innate and adaptive immune system‐associated signatures*

In sputum, signatures of downregulated genes after stimulation of macrophages by Interferonγ (IFNγ), lipopolysaccharide and Tumor Necrosis Factor/Prostaglandin E2/Toll‐like receptor 2 (TNF/PGE2/TLR2) activation were least enriched in FE (Table 4 and Table S2, signatures 86, 88 and 94, respectively).

In nasal brushings, there was a greater enrichment of a signature of upregulated genes derived from stimulating macrophages with TNF/PGE2/TLR2 (Table S2, signature 93) and IFNγ (Table S2, signature 93) and of downregulated genes after stimulation of macrophages with interleukin 4 (IL4) (Table S2, signature 88), reflecting a potential switch towards a non‐T2 status. A signature associated with monocyte activation (Table S2, signature 3) was also enriched to a greater extent in nasal brushings from FE compared with IE subjects (Table 4), reinforcing a switch to a non‐T2 phenotype.

In bronchial biopsies, there was a greater enrichment of a gene signature associated with the differentiation of the Innate Lymphoid Cell 3 (ILC3) subset of innate lymphoid cells (Table 4, Table S2 and signature 102). Signatures obtained from stimulation of lung epithelial cells by IL‐13 (Table S2, signature 99) were differentially enriched in FE, with the Th17 signature (Table S2, signature 39) downregulated in sputum and bronchial biopsies.

(iii) *Inflammation‐ and fibrosis‐associated signatures*

Three gene signatures from a PolyI:C‐exposed mouse model were differentially enriched in nasal brushings (upregulated) and sputum (downregulated) in FE (Table S2, signatures 62, 58 and 60, respectively). This may reflect evidence of early (24‐48 hours) viral infection in the nasal brushings but a reduced long‐term response to viral exposure in sputum cells. A fibrosis‐associated gene signature derived at the late phase (day 35) after bleomycin challenge in mice showed decreased enrichment in the sputum of FE (Table 4 and Supplementary Table S2, signature 76).

### 3.5 GSVA: comparing PFE versus PIE

**FIGURE 2:**
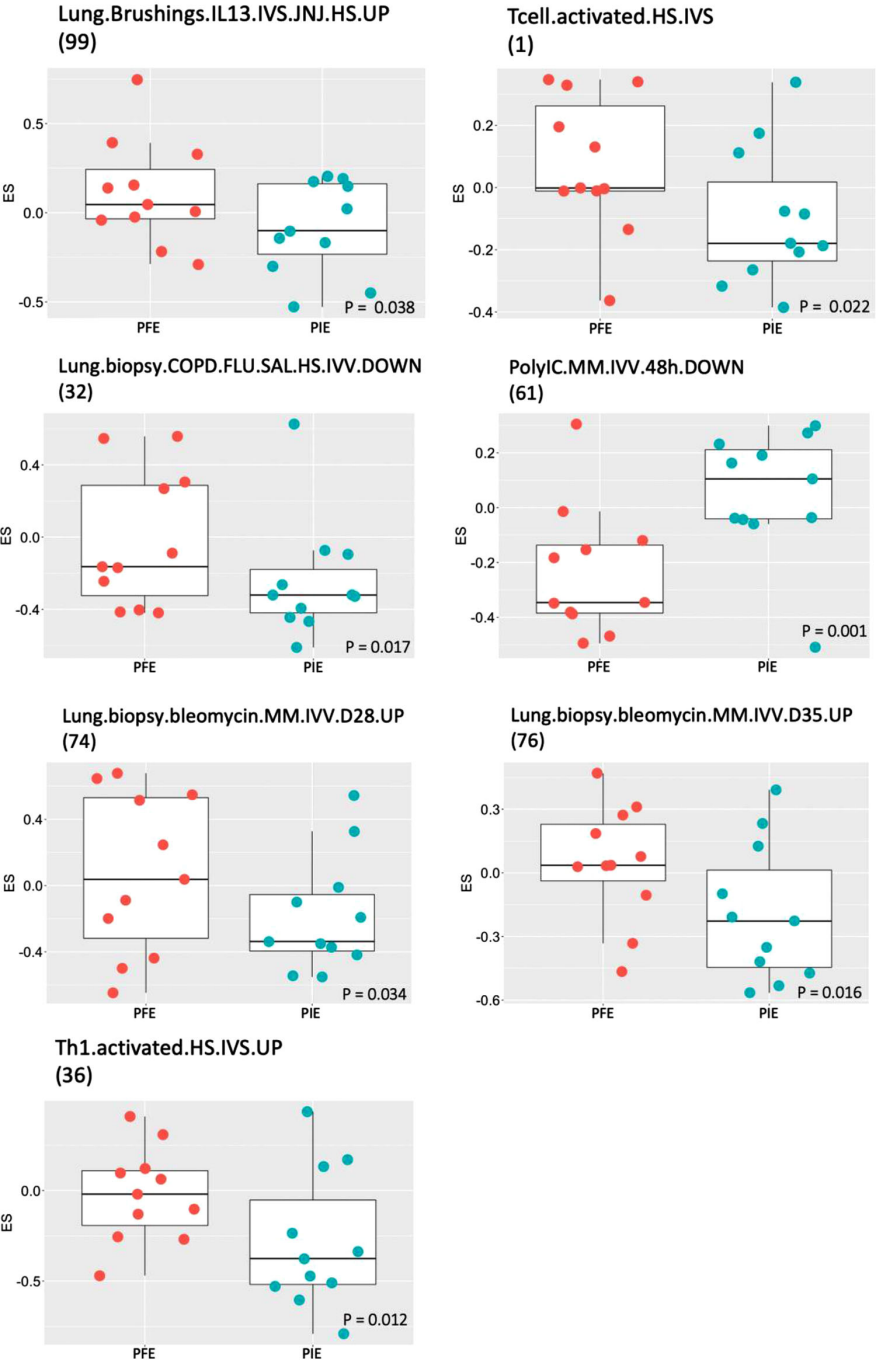
Expression scores (ES) of gene signatures in endobronchial biopsies of persistent frequent exacerbators (PFE) compared to persistent infrequent exacerbators (PIE). The gene signature number indicates the signature tabulated in Table S2. The *p* significance value is as indicated

Using GSVA, 27 gene signatures were differentially expressed in PFE compared to PIE in nasal brushings and bronchial biopsy samples (Figure 2 and Table 5). No signatures were differentially enriched in blood, sputum or bronchial brushings.

**TABLE 5:** Differences in enrichment scores by gene set variation analysis in persistent frequent exacerbators compared to persistent infrequent exacerbators

Pathway	Gene Signature	Signature number	Nasal brushings	Bronchial biopsy
T cells and cytokines Adaptive immune system	Lung.ASM.IL17a.healhty_mild.asthma.HS.IVS.UP	47	−0.433	
	Lung.ASM.IL17a.mild.asthma.HS.IVS.UP	48	−0.3921	
	Lung.Brushings.IL13.IVS.JNJ.HS.UP	99		0.2385
	siLP.ILC1.MM.UP	100	−0.2399	
	Tcell.activated.HS.IVS	1		0.2202
	Th1.activated.HS.IVS.UP	36		0.2762
	Tnaive.activated.HS.IVS.UP	40	−0.2472	
Autoimmune disease	PBMC.CD.HS.IVV.UP	21	−0.2337	
	PBMC.MS.HS.IVV.UP	15	−0.3126	
	PBMC.T1D.HS.IVV.UP	25	−0.3125	
	PBMC.UC.HS.IVV.UP	23	−0.2314	
Innate cells	Macrophage.GM_CSF.IL4.HS.IVS.DOWN	88	−0.34	
	Macrophage.GM_CSF.LPSc.HS.IVS.UP	89	−0.2251	
	Macrophage.GM_CSF.TNFa.HS.IVS.DOWN	92	−0.2709	
	Macrophage.GM_CSF.TNF.PGE2.P3C.HS.IVS.DOWN	94	−0.2032	
	Macrophage.GM_CSF.TNF.PGE2.P3C.HS.IVS.UP	93	−0.2621	
	Mast.cell.IgE.HS.IVS.UP	49	−0.3202	
	Monocyte.activated.HS.IVS	3	−0.2596	
	Neutrophil.activated.HS.IVS	4	−0.4232	
Lung fibrosis	Lung.biopsy.bleomycin.MM.IVV.D1.UP	65	−0.3815	
	Lung.biopsy.bleomycin.MM.IVV.D28.UP	74		0.3718
	Lung.biopsy.bleomycin.MM.IVV.D35.UP	76		0.2953
Steroid effects	Lung.ASM.asthma.Prednisolone.HS.IVV.UP	33	−0.3026	
	Lung.biopsy.COPD.FLU.SAL.HS.IVV.DOWN	32		0.3049
Viral INFLAMMATION	Lung.biopsy.PolyIC.MM.IVV.24 h.UP	58	−0.3184	
	Lung.biopsy.PolyIC.MM.IVV.48 h.DOWN	60		−0.3345
	Lung.biopsy.PolyIC.MM.IVV.96 h.DOWN	64	−0.5568	

*Note*: RED indicates higher enrichment score values in frequent exacerbators, and BLUE indicates lower enrichment score values.

Two gene signatures for IL‐17 from airway smooth muscle cells from mild asthmatic/healthy subjects or from mild asthmatic patients treated ex vivo with IL‐17α (Table 5 and Table S2, signatures 47 and 48, respectively) were downregulated in nasal brushings from PFE compared with PIE. A T2 signature obtained from stimulation of lung epithelial cells by IL‐13 (Table 5, Table S2, signature 99) was significantly enriched in bronchial biopsies of PFE. Gene signatures derived from the unique gene expression profile of activated T cells and Th1 cells (Table 5 and Table S2, signatures 1 and 36) were enriched in bronchial biopsies of PFE subjects.

Furthermore, two signatures from the late fibrotic phase mediated by lymphocytes and macrophages in a mouse model of bleomycin‐induced lung fibrosis (Table 5 and Table S2, signatures 74, 76) showed greater enrichment in bronchial biopsies of PFE subjects. This compared with the gene signature from the early phase of the same bleomycin‐challenged mouse model, which consists of an acute inflammatory response mainly mediated by neutrophils (Table 5, Table S2, signature 65), which showed decreased enrichment in nasal bushings of PFE subjects. In addition, a signature related to genes downregulated in COPD patients treated with combination therapy (Table S2, signature 32) was enriched in bronchial biopsies of PFE subjects (Table 5).

In contrast, other innate immune and autoimmune cell signatures, including those associated with viral infection, were significantly less enriched in the nasal brushings of PFE patients than in those of PIE subjects (Table 5). Overall, this suggests a switch from an immune cell‐driven inflammatory response in these subjects to potentially signatures more reflective of airway remodelling.

## 4 DISCUSSION

In the U‐BIOPRED severe asthma cohort,^22^ the FEs compared to IEs were more likely to be female, current smokers, report eczema and sinusitis more commonly, and were more symptomatic, with greater use of SABA and with more impaired quality of life. Multivariate regression analysis further refined these variables in highlighting being an ex‐smoker, high SABA use, presence of sinusitis and a lower age of onset of asthma. It is noted that the analysis highlights statistical associations; no causal relationships are inferred, and other correlated variables may represent better predictors. The results are descriptive and require independent validation. Certain features, such as sinusitis^23^, ^24^, ^25^ and smoking^11^, ^23^ ^,^ have been reported previously.

We then analysed the data in terms of the persistence of exacerbations at the 1‐year follow‐up visit. At the baseline visit, we classified 63% of the severe asthma cohort as being FEs with two or more exacerbations in the past year. At the 1‐year follow‐up, 63.6% of FEs were still FEs (labelled persistent FEs), while 61.3% of IEs remained IEs (persistent IEs), indicating that the FE phenotype as well as the IE phenotype was unstable. Persistent FE was more likely to be female, with an earlier onset of asthma, more commonly reported eczema, greater SABA use and OCS use, more symptoms and worse quality of life parameters. Contrary to what might be expected, FeNO and blood eosinophil counts were lower in the PFE group, and this could be explained by the greater OCS use in this group. Multivariate logistic regression analysis confirmed the association with the presence of eczema, high SABA use and higher mean ACQ5. Interestingly, being a current smoker was associated with a lower risk of PFE.

To understand the underlying pathways that may be associated with FEs, we compared the transcriptomic data of patients with FE to those with IE in various compartments, including blood, sputum, bronchial brushings and nasal brushings. We only found one differentially‐expressed gene between the two groups, with CEACAM5 expression in bronchial biopsies being more highly expressed in FE compared to IE. CEACAM5 is a member of the carcinoembryonic gene family, involved in cell signalling, cell proliferation, cell repair processes and maintenance of the bronchial epithelium, with evidence that this receptor is exploited by airway pathogens such as *Haemophilus influenzae*.^26^ In bronchial biopsies, CEACAM5 was the only one of two upregulated differentially‐expressed genes in the biopsies of severe asthma compared to nonsevere asthma.^14^ There were no significantly differentially expressed proteins between IE and FE or between PIE and FIE, but this does not negate the significance of the CEACAM5 transcript that was differentially expressed in bronchial biopsies. Any linked proteins to CEACAM5 may not have been covered by the Somalogic platform. In addition, the linked protein(s) may not be detectable in serum or sputum supernatants.

CEACAM5 is also one of the most highlyranked hub genes in the bronchial epithelium of patients with asthma.^27^ A signature containing CEACAM5 together with CD14 and TLR2 representing a response to bacterial infection has been described from an analysis of epithelial brushings and T‐cell transcriptomics from severe asthma patients.^28^ Its expression by immunohistochemistry is increased in the airway epithelium of patients with severe asthma.^29^ Therefore, CEACAM5 in the bronchial epithelium may be driving the increase in exacerbations noted in the FEs due to bacterial infection. However, CEACAM5 was not overexpressed in PFE compared to PIE. One potential reason for this may be related to the instability of the FE and IE phenotypes, as we have demonstrated, and the lack of power in detecting this transcript difference because of the lower number of follow‐up exacerbations. Using over 100 gene signatures selected to represent a range of immune cells and potential processes that may underpin frequent exacerbations of asthmatic subjects, we identified distinct cell and pathway activation states according to the compartment examined. There was a marked difference in pathway enrichment between FE and IE, which was dependent upon the compartment analysed. Nasal brushings showed enrichment of signatures associated with early (24‐48 hours) exposure to poly(I:C), a viral surrogate, in mice, which suggests that ongoing viral infection occurs in these subjects. This observation is supported by the recent finding that moderately‐severe asthmatic patients with high basal expression of the interferon gene in the nasal epithelium did not show an increase in the expression of this gene when challenged with rhinovirus; in addition, these subjects did not clear rhinovirus as well as those with lower basal levels of the interferon gene.^30^ However, a 72‐hour exposure to the poly(I:C) signature was not observed in nasal brushings; indeed, this signature was suppressed in sputum. This may reflect either a lack of chronicity or that infected cells do not migrate into the sputum at this later time‐point.

Nasal brushings also show enrichment for signatures associated with activated neutrophils and monocytes as well as for macrophages activated by IFNγ, LPS or TNF/PGE2/TLR2 exposure. Interestingly, the macrophage upsignatures were enriched in nasal brushings, while the downsignatures were less expressed in sputum. Together with the significant enrichment of the Th1‐associated multiple sclerosis peripheral blood mononuclear cell (PBMC) signature suggests a skewing of the immune response in FE towards a viral‐induced Th1 response. In contrast to expectation, we found mixed data regarding the enrichment of IL‐17/Th17‐associated signatures, with one reflecting the effect of IL‐17α‐stimulated airway smooth muscle cells being enriched in nasal brushings but other IL‐17 signatures being reduced in other compartments. These findings are in line with reports showing a lack of evidence for a role for Th‐17/IL‐17 pathways in asthma.^31^, ^32^, ^33^

GSVA of the five U‐BIOPRED compartments was also used to examine signatures that differentiated PFE from PIE. We found 27 differentially enriched GSVA signatures but only across nasal brushings and bronchial biopsies. There was enrichment of fibrotic signatures and a reduced steroid response signature compared with that seen with FE subjects in bronchial biopsies. For example, the signature for the downregulated gene response to combination therapy in COPD biopsies was enriched in bronchial biopsies of PFE subjects, while the signature reflecting the ability of prednisolone to upregulate ASM gene expression was reduced in nasal brushings from PFE subjects. In addition, there was a mixed Th1 and Th2 activated T‐cell response in bronchial biopsies of PFE subjects. The persistence of the enrichment of the IL‐13‐stimulated type 2 signature from bronchial brushings and an element of steroid resistance may underlie the pathways that might drive the need for higher steroid doses. We previously reported that the presence of tissue oeosinophilia, lower steroid responsiveness and a high T2 signature was associated with genes such as CD44^14^ that may play a critical role in airway remodelling and the recruitment of specific immune cells into the airways of PFEs.

In contrast, most immune cell signatures were either not differentially‐expressed between PFE and PIE or had a significantly lower ES in nasal brushings of PFEs. There were also reduced expression scores for poly(I:C)/viral response signatures in both nasal brushings and bronchial biopsies. Thus, PFE patients may have a greater tendency for remodelling to occur in the absence of clear immune cell recruitment. However, we did not examine the expression of all immune cells and their activation states, which could confirm their important role in PFE.

One interesting observation is the lower risk of persistent exacerbations in severe asthmatic patients who were current smokers compared to nonsmokers and ex‐smokers. We did not find evidence of an increase in the type 1 response that could suppress type 2 inflammation, which could be the basis for exacerbations. In our previous analysis, we reported that GSVA of the bronchial epithelial cell transcriptome from current smokers showed enrichment of xenobiotic metabolism, oxidative stress and endoplasmic reticulum stress compared to the other groups.^34^ We speculate that these pathways may protect to some extent from recurrent exacerbations. A plausible explanation would be that smoking through these or other unknown protective mechanisms on airway inflammation would provide a survivorship bias.

One weakness of the study is that we only used the MARS questionnaire as a measure of adherence that indicated good adherence to medication, particularly inhaled and oral corticosteroid therapy. This should have been backed by a direct assessment of prescriptions provided by the participant's doctor.

In summary, multivariate regression analysis identified being an ex‐smoker, high SABA use, presence of sinusitis and a lower age of onset of asthma as differentiating FE from IE in the U‐BIOPRED cohort. On the other hand, the subgroup of FE patients with PFE compared with PIE had more uncontrolled asthma needing SABA and OCS use, a greater presence of eczema, and higher ACQ5 scores. CEACAM5 was the only differentially expressed gene that differentiated FE from IE, but this was not the case for PFE compared with PIE. Persistence of frequent exacerbations in PFE was associated with increased T1‐cell activation, IL‐13 overexpression and fibrosis pathways, with a reduced response to viral infections, providing some insight into the driving mechanism for the persistence of frequent exacerbations.

## CONFLICTS OF INTERESTS

Dr Singer reports honoraria for lectures and presentations from Novartis Pharma Switzerland and Vertex Pharmaceuticals Switzerland, outside the submitted work. Dr Shaw has received speaker fees from Chiesi and Astra Zeneca and advisory board fees from Adherium, Nuvoair, Astra Zeneca and Chiesi. Dr Loza is employed by and own stock in Johnson & Johnson. Dr. SE Dahlén reports personal fees from AstraZeneca, Cayman Chemicals, GSK, Novartis, Regeneron, Sanofi and Teva for consultancies outside the submitted work. Drs Auffray, Demulder and Lefaudeux report grants from the IMI (U‐BIOPRED n°115010 and eTRIKS n°115446). Dr Fowler has received grants from Boehringer Ingelheim and fees from Chiesi, outside of the current work. Dr Djukanovic reports receiving fees for lectures at symposia organised by Novartis, AstraZeneca and TEVA, consultation for TEVA and Novartis as members of advisory boards, and participation in a scientific discussion about asthma organised by GlaxoSmithKline. He is a co‐founder and current consultant and has shares in Synairgen, a University of Southampton spin out company. Dr Sterk reports grants from public–private funding by the Innovative Medicines Initiative (IMI), outside the submitted work. Dr Chung has received honoraria for participating in Advisory Board meetings of GSK, AZ, Roche, Novartis, Merck, BI, TEVA and Shionogi regarding treatments for asthma, chronic obstructive pulmonary disease and chronic cough and has also been remunerated for speaking engagements. The other authors have no disclosures in relation to this work. U‐BIOPRED was supported by an Innovative Medicines Initiative Joint Undertaking (No. 115010), resources from the European Union's Seventh Framework Programme (FP7/2007‐2013) and EFPIA companies’ in‐kind contribution (www.imi.europa.eu). KFC and IMA are funded by UK Research and Innovation (UKRI). KFC is an Emeritus Senior Investigator of the UK National Institute for Health Research (NIHR). CEW is funded by the Swedish Heart Lung Foundation (HLF 20180290, HLF 20200693). We acknowledge the contribution of the whole U‐BIOPRED team as listed in the Supporting Information repository file.

## Supporting information

SUPPORTING INFORMATION
