# Supplementary material for: Clinical and transcriptomic features of persistent exacerbation‐prone severe asthma in U‐BIOPRED cohort
Source: Clin Transl Med. 2022 Apr 26;12(4):e816. doi: 10.1002/ctm2.816 (PMC9043117; doi:10.1002/ctm2.816)
Supplement: Supplementary file 1 — SUPPORTING INFORMATION [file CTM2-12-e816-s001.docx]

**Supplementary material**

**Clinical and transcriptomic features of persistent exacerbation-prone**

**severe asthma**

Uruj Hoda, Stelios Pavlidis, Aruna T. Bansal, Kentaro Takahashi, Sile Hu, Francois Ng Kee Kwong , Christos Rossios, Kai Sun, Pankaj Bhavsar, Matthew Loza, Fred Baribaud, Pascal Chanez, Stephen J Fowler, Ildiko Horvath, Paolo Montuschi, Florian Singer, Jacek Musial, Barbro Dahlen, Norbert Krug, Thomas Sandstrom, Dominic E. Shaw, Rene Lutter, Louise J. Fleming, Peter H. Howarth, Massimo Caruso, Ana R Sousa, Julie Corfield, Charles Auffray, Bertrand De Meulder, Diane Lefaudeux, Sven-Erik Dahlen , Ratko Djukanovic, Peter J Sterk, Yike Guo, Ian M. Adcock, Kian Fan Chung on behalf of the U-BIOPRED study group#

***Contents***

**Fig S1**

**Table S1**

**Table S2**

**List of the U-BIOPRED Consortium project team members**

**Supplementary Fig S1**


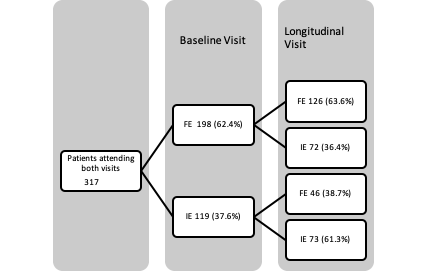


Number of frequent exacerbators (FE) and infrequent exacerbators (IE) at baseline visit and of these exacerbators at a longitudinal visit at one year when the exacerbation status weas redetermined on the basis of exacerbations during the one year follow-up. The persistent FE are part of the FE group at baseline visit, while the persistent IE are part of the IE group at baseline visit.

**Supplementary Table S1. Characteristics of patients according to provision of different samples**

|  | **Blood** | **Sputum** | **Nasal Brushings** | **Bronchial Biopsy** | **Bronchial Brushings** |
| --- | --- | --- | --- | --- | --- |
| **N** | 334 | 84 | 42 | 53 | 67 |
| **Sex: % Female*** | 204 (61.1%) | 51 (58.3%) | 22 (52.52%) | 28 (52.83) | 32 (47.76%) |
| **Age^#^** | 51.98 (13.76) | 53.54 (11.82) | 50.14 (14.11) | 50 (12.72%) | 49.52 (13.03) |
| **Body Mass Index (kg/m^2^)^#^** | 29.18 (6.18) | 28.30 (5.19) | 31.82 (6.23) | 29.62 (6.05) | 30.25 (6.28) |
| **Smoking Status*** |  |  |  |  |  |
| ***Current*** | 38 (11.3%) | 8 (9.52%) | 3 (7.14%) | 7 (13.21%) | 6 (12.76%) |
| ***ex-smoker*** | 88 (26.3%) | 29 (34.52%) | 13 (30.95%) | 12 (22.64%) | 21 (31.34%) |
| ***non-smoker*** | 208 (62.3%) | 47 (55.95%) | 26 (61.90%) | 34 (54.15%) | 40 (59.70%) |
| **Pack-Years^$^** | 12.88 (4 - 22.84) | 11 (2.25-20.5) | 23.13 (23.54) | 18.42 (16.01) | 19.11 (19.80) |
| **Atopic*** | 239 (71.47%) | 62 (73.17%) | 31 (73.81%) | 36 (69.23%) | 48 (72.72%) |
| **Exacerbations^#^** | 3 (2.23) | 2.92 (1.93) | 2.72 (1.66) | 3.18 (2.04) | 2.79 (1.59) |
| **ACQ5^#^** | 2.26 (1.17) | 2.18 (1.28) | 1.79 (1.09) | 1.97 (1.16) | 1.97 (1.09) |
| **Allergic Rhinitis*** | 184 (55.21%) | 31 (41.89%) | 20 (51.28%) | 27 (54%) | 33 (53.22) |
| **Diabetes*** | 35 (10.57%) | 11 (13.41%) | 20 (48.78%) | 1 (1.92%) | 1 (1.56%) |
| **Eczema*** | 117 (35.16%) | 26 (32.09%) | 23 (56.09%) | 26 (50%) | 34 (53.12) |
| **GORD*** | 170 (50.98%) | 38 (48.10%) | 29 (70.73%) | 29 (56.86%) | 38 (60.31%) |
| **Nasal Polyps*** | 106 (31.81%) | 30 (37.03%) | 9 (23.07%) | 19 (37.25%) | 5 (40.98%) |
| **Sinusitis*** | 112 (33.65%) | 24 (30.38%) | 10 (25%) | 15 (29.41%) | 20 (31.74%) |
| **Age of onset^$^** | 27 (10-43) | 26 (7-43) | 23.53 (19.35%) | 22.19 (20.19%) | 23.32 (19.24) |
| **FEV_1_(L)^#^** | 1.95 (0.80) | 1.87 (0.83) | 2.31 (0.75) | 2.19 (0.75) | 2.28 (0.73) |
| **FEV_1_(% predicted)^#^** | 67.23 (21.21) | 63.02 (20.66) | 77.00 (21.37) | 71.34 (19.25%) | 73.22 (20.39) |
| **FeNO (ppb)^#^** | 36.41 (31.03) | 38.96 (37.10) | 33.59 (26.42%) | 35.35 (28.34%) | 37.90 (28.86) |
| **Blood Neutrophils (10^3^/uL)^#^** | 5.22 (2.20) | 5.44 (2.39) | 5.26 (2.13) | 5.33 (2.45) | 5.36 (2.48) |
| **Blood Eosinophils (10^3^/uL)^$^** | 0.2 (0.1-0.4) | 0.3 (0.16 - 0.44) | 0.2 (0.1 - 0.38) | 0.2 (0.1 - 03) | 0.2 (0.1 - 0.3) |
| **Sputum (% Neutrophils)^#^** | 51.9 (25.3) | 58.96 (25.71) | 54.70 (20.18) | 52.03 (22.42%) | 53.69 (22.47) |
| **Sputum (% Eosinophils)^$^** | 2.9 (0.94 - 16.13) | 3.51 (0.38 - 18.45) | 1.31 (0.32 - 8.27) | 2.31 (0.58 - 7.5) | 1.42 (0.38 - 15.99) |
| * Number (percentage); ^#^ Mean (SD); ^$^ Median (Interquartile range) | | | | | |
| ACQ: Asthma Control Questionnaire; FeNO: Fractional level of nitric oxide in exhaled breath; FEV1 : Forced expiratory volume in one second; GORD : Gastro-oesophageal reflux disease; ppb: parts per billion. | | | | | |
|  | | | | | |

**Supplementary Table S2. Transcriptomic signatures used for gene-set variation analysis.**

|  | **Transcriptomic signature** | **Condition/activation** | **type** | **Cell type/tissue** | **species** | **reference** |
| --- | --- | --- | --- | --- | --- | --- |
| 1 | Tcell.activated.HS.IVS | Th1: IL12, IFN-delta, anti-IL4 - Th2: anti-IL12, anti-IFN-delta, IL4, then anti-CD3 and anti-CD28. | IVS | Tcell | HS | <http://www.ncbi.nlm.nih.gov/pubmed/15789058> |
| 2 | Bcell.activated.HS.IVS | isolated with MACS® CD138 microbeads and CD19 microbeads | IVS | Bcell | HS | http://www.ncbi.nlm.nih.gov/pubmed/15789058 |
| 3 | Monocyte.activated.HS.IVS | isolated with MACS^®^ CD14 Microbeads | IVS | Monocyte | HS | <http://www.ncbi.nlm.nih.gov/pubmed/15789058> |
| 4 | NKcell.activated.HS.IVS | RosetteSep™ NK-cell enrichment cocktail and further purified by CD2 microBeads | IVS | NKcell | HS | http://www.ncbi.nlm.nih.gov/pubmed/15789058 |
| 5 | Dentritic.activated.HS.IVS | IL4, and 67 | IVS | Dentritic | HS | http://www.ncbi.nlm.nih.gov/pubmed/15789058 |
| 6 | Neutrophil.activated.HS.IVS | Heparanized blood mixed with an equal volume of dextran/saline solution. | IVS | Neutrophil | HS | http://www.ncbi.nlm.nih.gov/pubmed/15789058 |
| 7 | Neutrophil.nas.brushings.HS |  |  | neutrophils.nasal.brushings.Hansel | HS | Malkov, Hansel et al, unpublished |
| 8 | Lung.biopsy.HDM.Rhesus.IVV.UP | HDM | EXVIVO | lung biopsy | Rhesus | <http://www.ncbi.nlm.nih.gov/pubmed/21819959> |
| 9 | Lung.brushings.asthma.HS.IVV.UP | asthma | EXVIVO | lung brushings | HS | <http://www.ncbi.nlm.nih.gov/pubmed/17898169> |
| 10 | Lung.brushings.asthma.HS.IVIVO.DOWN | asthma | EXVIVO | lung brushings | HS | <http://www.ncbi.nlm.nih.gov/pubmed/17898169> |
| 11 | Lung.brushings.FLU.asthma.HS.IVV.UP | fluticasone (FLU) | EXVIVO | bronchial epithelia | HS | <http://www.ncbi.nlm.nih.gov/pubmed/17898169> |
| 12 | Lung.brushings.FLU.asthma.HS.IVV.DOWN | fluticasone (FLU) | EXVIVO | bronchial epithelia | HS | <http://www.ncbi.nlm.nih.gov/pubmed/17898169> |
| 13 | Lung.biopsy.Th2high.asthma.HS.IVV.UP | Th2 high/low asthma | EXVIVO | lung biopsy | HS | <http://www.ncbi.nlm.nih.gov/pubmed/21187436> |
| 14 | Lung.biopsy.Th2high.asthma.HS.IVV.DOWN | Th2 high/low asthma | EXVIVO | lung biopsy | HS | http://www.ncbi.nlm.nih.gov/pubmed/21187436 |
| 15 | PBMC.MS.HS.IVV.UP | Multiple sclerosis (MS) | EXVIVO | peripheral blood | HS | <http://www.ncbi.nlm.nih.gov/pubmed/23190644> |
| 16 | PBMC.MS.HS.IVV.DOWN | Multiple sclerosis (MS) | EXVIVO | peripheral blood | HS | http://www.ncbi.nlm.nih.gov/pubmed/23190644 |
| 17 | PBMC.SLE.HS.IVV.UP | Systemic lupus erythematosus (SLE) | EXVIVO | peripheral blood | HS | http://www.ncbi.nlm.nih.gov/pubmed/23190644 |
| 18 | PBMC.SLE.HS.IVV.DOWN | Systemic lupus erythematosus (SLE) | EXVIVO | peripheral blood | HS | http://www.ncbi.nlm.nih.gov/pubmed/23190644 |
| 19 | PBMC.JRA.HS.IVV.UP | Juvenile rheumatoid arthritis (JRA) | EXVIVO | peripheral blood | HS | http://www.ncbi.nlm.nih.gov/pubmed/23190644 |
| 20 | PBMC.JRA.HS.IVV.DOWN | Juvenile rheumatoid arthritis (JRA) | EXVIVO | peripheral blood | HS | http://www.ncbi.nlm.nih.gov/pubmed/23190644 |
| 21 | PBMC.CD.HS.IVV.UP | Crohn’s disease (CD) | EXVIVO | peripheral blood | HS | http://www.ncbi.nlm.nih.gov/pubmed/23190644 |
| 22 | PBMC.CD.HS.IVV.DOWN | Crohn’s disease (CD) | EXVIVO | peripheral blood | HS | http://www.ncbi.nlm.nih.gov/pubmed/23190644 |
| 23 | PBMC.UC.HS.IVV.UP | Ulcerative colitis (UC) | EXVIVO | peripheral blood | HS | http://www.ncbi.nlm.nih.gov/pubmed/23190644 |
| 24 | PBMC.UC.HS.IVV.DOWN | Ulcerative colitis (UC) | EXVIVO | peripheral blood | HS | http://www.ncbi.nlm.nih.gov/pubmed/23190644 |
| 25 | PBMC.T1D.HS.IVV.UP | Type 1 diabetes (T1D) | EXVIVO | peripheral blood | HS | http://www.ncbi.nlm.nih.gov/pubmed/23190644 |
| 26 | PBMC.T1D.HS.IVV.DOWN | Type 1 diabetes (T1D) | EXVIVO | peripheral blood | HS | http://www.ncbi.nlm.nih.gov/pubmed/23190644 |
| 27 | CD4T.RA.HS.IVV.UP | Rheumatoid Arthritis (RA) | EXVIVO | peripheral blood | HS | http://www.ncbi.nlm.nih.gov/pubmed/22532634 |
| 28 | Th17.activated.HS.IVS.UP.PMID22715389 | activated | EXVIVO | peripheral blood | HS | <http://www.ncbi.nlm.nih.gov/pubmed/22715389> |
| 29 | PAXgene.H1N1.HS.IVV.UP | H1N1 - trivalent influenza vaccine | EXVIVO | peripheral blood | HS | <http://www.ncbi.nlm.nih.gov/pubmed/21357945> |
| 30 | PAXgene.H1N1.HS.IVV.DOWN | H1N1 - trivalent influenza vaccine | EXVIVO | peripheral blood | HS | <http://www.ncbi.nlm.nih.gov/pubmed/21357945> |
| 31 | Lung.biopsy.COPD.FLU.SAL.HS.IVV.UP | COPD fluticasone (FLU) salmeterol (SAL) | EXVIVO | lung biopsy | HS | http://www.ncbi.nlm.nih.gov/pubmed/23925644 |
| 32 | Lung.biopsy.COPD.FLU.SAL.HS.IVV.DOWN | COPD. fluticasone.salmeterol | EXVIVO | lung biopsy | HS | http://www.ncbi.nlm.nih.gov/pubmed/23925644 |
| 33 | Lung.ASM.asthma.Prednisolone.HS.IVV.UP | asthma - Airway Smooth Muscle (ASM) prednisolone | EXVIVO | lung biopsy airway smooth muscle (ASM) | HS | <http://www.ncbi.nlm.nih.gov/pubmed/23491407> |
| 34 | PBMC.SLE.IFNa.HS.IVV_IVS.UP | Systemic lupus erythematosus (SLE) and PBMC | EXVIVO_IVS | peripheral blood | HS | <http://www.ncbi.nlm.nih.gov/pubmed/12642603> |
| 35 | PBMC.SLE.IFNa.HS.IVV_IVS.DOWN | Systemic lupus erythematosus (SLE) and PBMC | EXVIVO_IVS | peripheral blood | HS | <http://www.ncbi.nlm.nih.gov/pubmed/12642603> |
| 36 | Th1.activated.HS.IVS.UP | anti-CD3, soluble anti-CD28, in the presence of IL-12, IL-2 and anti–IL-4 | IVS | peripheral blood | HS | <http://www.ncbi.nlm.nih.gov/pubmed/23870669> |
| 37 | Th2.activated.HS.IVS.UP | IL-4, IL-2 and anti-IL-12, and anti–IFN-g | IVS | peripheral blood | HS | <http://www.ncbi.nlm.nih.gov/pubmed/23870669> |
| 38 | Th2.activated.HS.IVS.DOWN | IL-4, IL-2 and anti-IL-12, and anti–IFN-g | IVS | peripheral blood | HS | <http://www.ncbi.nlm.nih.gov/pubmed/23870669> |
| 39 | Th17.activated.HS.IVS.UP.PMID23870669 | TGF-β, IL-1β, IL-6, IL-21, and IL-23 | IVS | peripheral blood | HS | <http://www.ncbi.nlm.nih.gov/pubmed/23870669> |
| 40 | Tnaive.activated.HS.IVS.UP | total CD4^+^ T cells were labeled with Pacific Blue™ anti-human CD4, PE anti-human CCR7, FITC anti-human CD45RO and APC anti-human CD45RA | IVS | peripheral blood | HS | <http://www.ncbi.nlm.nih.gov/pubmed/23870669> |
| 41 | Tnaive.activated.HS.IVS.DOWN | total CD4^+^ T cells were labeled with Pacific Blue™ anti-human CD4, PE anti-human CCR7, FITC anti-human CD45RO and APC anti-human CD45RA | IVS | peripheral blood | HS | <http://www.ncbi.nlm.nih.gov/pubmed/23870669> |
| 42 | Treg.activated.HS.IVS.UP | activated | IVS | peripheral blood | HS | <http://www.ncbi.nlm.nih.gov/pubmed/23870669> |
| 43 | Treg.activated.HS.IVS.DOWN | activated | IVS | peripheral blood | HS | <http://www.ncbi.nlm.nih.gov/pubmed/23870669> |
| 44 | CD4.severe.asthma.HS.IVV.UP | Severe Asthma | EXVIVO | peripheral blood | HS | <http://www.ncbi.nlm.nih.gov/pubmed/21917308> |
| 45 | CD8.severe.asthma.HS.IVV.UP | Severe Asthma | EXVIVO | peripheral blood | HS | <http://www.ncbi.nlm.nih.gov/pubmed/21917308> |
| 46 | CD8.severe.asthma.HS.IVV.DOWN | Severe Asthma | EXVIVO | peripheral blood | HS | <http://www.ncbi.nlm.nih.gov/pubmed/21917308> |
| 47 | Lung.ASM.IL17a.healhty_mild.asthma.HS.IVS.UP | IL17 Healthy & Mild Asthma gene targets | IVS | lung biopsy airway smooth muscle (ASM) | HS | <http://www.ncbi.nlm.nih.gov/pubmed/24393021> |
| 48 | Lung.ASM.IL17a.mild.asthma.HS.IVS.UP | IL17, Mild Asthmatics | IVS | lung biopsy airway smooth muscle (ASM) | HS | <http://www.ncbi.nlm.nih.gov/pubmed/24393021> |
| 49 | Mast.cell.IgE.HS.IVS.UP | IgE | IVS | Human umbilical cord blood | HS | <http://www.ncbi.nlm.nih.gov/pubmed/16911805> |
| 50 | Mast.cell.Hansel |  |  | neutrophils.nasal.brushings.Hansel | HS | Malkov, Hansel et al, unpublished |
| 51 | Sputum.asthma.HS.UP | Mixed Granulocytic Asthma | EXVIVO | Sputum | HS | <http://www.ncbi.nlm.nih.gov/pubmed/24582314> |
| 52 | PBMC.asthma.GC.HS.IVS | Asthma,IL-1β, TNF-α, with or without GC | EXVIVO | PBMC | HS | <http://www.ncbi.nlm.nih.gov/pmc/articles/PMC1253826/> |
| 53 | BAL.eosinophils.WLAC.HS.IVV.UP | Mild Asthma, Whole Lung Allergen Challenge (WLAC) | EXVIVO | Bronchoalveolar lavage (BAL) | HS | <http://www.ncbi.nlm.nih.gov/pubmed/23844029> |
| 54 | Eosinophils.nasal.brushings.HS.Hansel |  |  | Eosinophils.nasal.brushings.Hansel | HS | Malkov, Hansel et al, unpublished |
| 55 | Lung.biopsy.PolyIC.MM.IVV.2h.UP | polyIC | EXVIVO | lung biopsy | MM, BALB/cJ mice | <http://www.ncbi.nlm.nih.gov/pubmed/22990623> |
| 56 | Lung.biopsy.PolyIC.MM.IVV.6h.UP | polyIC | EXVIVO | lung biopsy | MM, BALB/cJ mice | <http://www.ncbi.nlm.nih.gov/pubmed/22990623> |
| 57 | Lung.biopsy.PolyIC.MM.IVV.6h.DOWN | polyIC | EXVIVO | lung biopsy | MM, BALB/cJ mice | <http://www.ncbi.nlm.nih.gov/pubmed/22990623> |
| 58 | Lung.biopsy.PolyIC.MM.IVV.24h.UP | polyIC | EXVIVO | lung biopsy | MM, BALB/cJ mice | <http://www.ncbi.nlm.nih.gov/pubmed/22990623> |
| 59 | Lung.biopsy.PolyIC.MM.IVV.24h.DOWN | polyIC | EXVIVO | lung biopsy | MM, BALB/cJ mice | <http://www.ncbi.nlm.nih.gov/pubmed/22990623> |
| 60 | Lung.biopsy.PolyIC.MM.IVV.48h.UP | polyIC | EXVIVO | lung biopsy | MM, BALB/cJ mice | <http://www.ncbi.nlm.nih.gov/pubmed/22990623> |
| 61 | Lung.biopsy.PolyIC.MM.IVV.48h.DOWN | polyIC | EXVIVO | lung biopsy | MM, BALB/cJ mice | <http://www.ncbi.nlm.nih.gov/pubmed/22990623> |
| 62 | Lung.biopsy.PolyIC.MM.IVV.72h.UP | polyIC | EXVIVO | lung biopsy | MM, BALB/cJ mice | <http://www.ncbi.nlm.nih.gov/pubmed/22990623> |
| 63 | Lung.biopsy.PolyIC.MM.IVV.96h.UP | polyIC | EXVIVO | lung biopsy | MM, BALB/cJ mice | <http://www.ncbi.nlm.nih.gov/pubmed/22990623> |
| 64 | Lung.biopsy.PolyIC.MM.IVV.96h.DOWN | polyIC | EXVIVO | lung biopsy | MM, BALB/cJ mice | <http://www.ncbi.nlm.nih.gov/pubmed/22990623> |
| 65 | Lung.biopsy.bleomycin.MM.IVV.D1.UP | Bleomycin | EXVIVO | lung biopsy | MM,C57BL6/J | <http://www.ncbi.nlm.nih.gov/pubmed/23565148> |
| 66 | Lung.biopsy.bleomycin.MM.IVV.D2.UP | Bleomycin | EXVIVO | lung biopsy | MM,C57BL6/J | <http://www.ncbi.nlm.nih.gov/pubmed/23565148> |
| 67 | Lung.biopsy.bleomycin.MM.IVV.D2.DOWN | Bleomycin | EXVIVO | lung biopsy | MM,C57BL6/J | <http://www.ncbi.nlm.nih.gov/pubmed/23565148> |
| 68 | Lung.biopsy.bleomycin.MM.IVV.D7.UP | Bleomycin | EXVIVO | lung biopsy | MM,C57BL6/J | <http://www.ncbi.nlm.nih.gov/pubmed/23565148> |
| 69 | Lung.biopsy.bleomycin.MM.IVV.D7.DOWN | Bleomycin | EXVIVO | lung biopsy | MM,C57BL6/J | <http://www.ncbi.nlm.nih.gov/pubmed/23565148> |
| 70 | Lung.biopsy.bleomycin.MM.IVV.D14.UP | Bleomycin | EXVIVO | lung biopsy | MM,C57BL6/J | <http://www.ncbi.nlm.nih.gov/pubmed/23565148> |
| 71 | Lung.biopsy.bleomycin.MM.IVV.D14.DOWN | Bleomycin | EXVIVO | lung biopsy | MM,C57BL6/J | <http://www.ncbi.nlm.nih.gov/pubmed/23565148> |
| 72 | Lung.biopsy.bleomycin.MM.IVV.D21.UP | Bleomycin | EXVIVO | lung biopsy | MM,C57BL6/J | <http://www.ncbi.nlm.nih.gov/pubmed/23565148> |
| 73 | Lung.biopsy.bleomycin.MM.IVV.D21.DOWN | Bleomycin | EXVIVO | lung biopsy | MM,C57BL6/J | <http://www.ncbi.nlm.nih.gov/pubmed/23565148> |
| 74 | Lung.biopsy.bleomycin.MM.IVV.D28.UP | Bleomycin | EXVIVO | lung biopsy | MM,C57BL6/J | <http://www.ncbi.nlm.nih.gov/pubmed/23565148> |
| 75 | Lung.biopsy.bleomycin.MM.IVV.D28.DOWN | Bleomycin | EXVIVO | lung biopsy | MM,C57BL6/J | <http://www.ncbi.nlm.nih.gov/pubmed/23565148> |
| 76 | Lung.biopsy.bleomycin.MM.IVV.D35.UP | Bleomycin | EXVIVO | lung biopsy | MM,C57BL6/J | <http://www.ncbi.nlm.nih.gov/pubmed/23565148> |
| 77 | Macrophage.FP.HS.UP | Fluticasone Propionate (FP) | IVS | PBMC | HS | <http://www.ncbi.nlm.nih.gov/pubmed/24395918> |
| 78 | Macrophage.FP.HS.DOWN | Fluticasone Propionate (FP) | IVS | PBMC | HS | <http://www.ncbi.nlm.nih.gov/pubmed/24395918> |
| 79 | GCGS.DEX.HS.UP | dexamethasone (DEX) | IVS | Glucocorticoid Gene Set (GCGS),Lymphoblasts, lung | HS, MM | <http://www.ncbi.nlm.nih.gov/pubmed/25192440> |
| 80 | GCGS.DEX.HS.DOWN | dexamethasone (DEX) | IVS | Glucocorticoid Gene Set (GCGS), Lymphoblasts, lung | HS, MM | <http://www.ncbi.nlm.nih.gov/pubmed/25192440> |
| 81 | DGGS.DEX.HS.UP | dexamethasone (DEX) | IVS | Developmental Glucocorticoid Gene Set (DGGS) | HS, MM | <http://www.ncbi.nlm.nih.gov/pubmed/25192440> |
| 82 | DGGS.DEX.HS.DOWN | dexamethasone (DEX) | IVS | Developmental Glucocorticoid Gene Set (DGGS) | HS, MM | <http://www.ncbi.nlm.nih.gov/pubmed/25192440> |
| 83 | Macrophage.GM_CSF.HS.IVS.UP | GM_CSF | IVS | Buffy coats from healthy donors | HS | <http://www.ncbi.nlm.nih.gov/pubmed/24530056> |
| 84 | Macrophage.GM_CSF.HS.IVS.DOWN | GM_CSF | IVS | Buffy coats from healthy donors | HS | http://www.ncbi.nlm.nih.gov/pubmed/24530056 |
| 85 | Macrophage.GM_CSF.IFNg.HS.IVS.UP | IFNg | IVS | Buffy coats from healthy donors | HS | http://www.ncbi.nlm.nih.gov/pubmed/24530056 |
| 86 | Macrophage.GM_CSF.IFNg.HS.IVS.DOWN | IFNg | IVS | Buffy coats from healthy donors | HS | http://www.ncbi.nlm.nih.gov/pubmed/24530056 |
| 87 | Macrophage.GM_CSF.IL4.HS.IVS.UP | IL4 | IVS | Buffy coats from healthy donors | HS | http://www.ncbi.nlm.nih.gov/pubmed/24530056 |
| 88 | Macrophage.GM_CSF.IL4.HS.IVS.DOWN | IL4 | IVS | Buffy coats from healthy donors | HS | http://www.ncbi.nlm.nih.gov/pubmed/24530056 |
| 89 | Macrophage.GM_CSF.LPSc.HS.IVS.UP | LPSc | IVS | Buffy coats from healthy donors | HS | http://www.ncbi.nlm.nih.gov/pubmed/24530056 |
| 90 | Macrophage.GM_CSF.LPSc.HS.IVS.DOWN | LPSc | IVS | Buffy coats from healthy donors | HS | http://www.ncbi.nlm.nih.gov/pubmed/24530056 |
| 91 | Macrophage.GM_CSF.TNFa.HS.IVS.UP | TNFa | IVS | Buffy coats from healthy donors | HS | http://www.ncbi.nlm.nih.gov/pubmed/24530056 |
| 92 | Macrophage.GM_CSF.TNFa.HS.IVS.DOWN | TNFa | IVS | Buffy coats from healthy donors | HS | http://www.ncbi.nlm.nih.gov/pubmed/24530056 |
| 93 | Macrophage.GM_CSF.TNFa.PGE2.P3C.HS.IVS.UP | TNFa.PGE2.P3C | IVS | Buffy coats from healthy donors | HS | http://www.ncbi.nlm.nih.gov/pubmed/24530056 |
| 94 | Macrophage.GM_CSF.TNFa.PGE2.P3C.HS.IVS.DOWN | TNFa.PGE2.P3C | IVS | Buffy coats from healthy donors | HS | http://www.ncbi.nlm.nih.gov/pubmed/24530056 |
| 95 | Macrophage.GM_CSF.upLPS.HS.IVS.UP | upLPS | IVS | Buffy coats from healthy donors | HS | http://www.ncbi.nlm.nih.gov/pubmed/24530056 |
| 96 | Macrophage.GM_CSF.upLPS.HS.IVS.DOWN | upLPS | IVS | Buffy coats from healthy donors | HS | http://www.ncbi.nlm.nih.gov/pubmed/24530056 |
| 97 | LUNG.ASM.DEX.HS.UP | dexamethasone (DEX) | IVS | LUNG Airway Smooth Muscle (ASM) | HS | <http://www.ncbi.nlm.nih.gov/pubmed/24926665> |
| 98 | LUNG.ASM.DEX.HS.DOWN | dexamethasone (DEX) | IVS | LUNG Airway Smooth Muscle (ASM) | HS | <http://www.ncbi.nlm.nih.gov/pubmed/24926665> |
| 99 | Lung.Brushings.IL13.IVS.JNJ.HS.UP | IL13 - lung epithelia | IVS | Lung brushings | HS | ADEPT |
| 100 | siLP.ILC1.MM.UP | ILC1 genes uniquely upregulated at least 2-fold compared to ILC2, ILC3, and LTi-like | IVS | small intestine lamina propria | MM | <http://www.ncbi.nlm.nih.gov/pubmed/25621825> |
| 101 | siLP.ILC2.MM.UP | ILC2 genes uniquely upregulated at least 2-fold compared to compared to ILC1, ILC3, and LTi-like | IVS | small intestine lamina propria | MM | <http://www.ncbi.nlm.nih.gov/pubmed/25621825> |
| 102 | siLP.ILC3.MM.UP | ILC3 genes uniquely upregulated at least 2-fold compared to compared to ILC1, ILC2, and LTi-like | IVS | small intestine lamina propria | MM | <http://www.ncbi.nlm.nih.gov/pubmed/25621825> |
| 103 | siLP.Lti.MM.UP | LTi-like genes uniquely upregulated at least 2-fold compared to compared to ILC1, ILC2, and ILC3 | IVS | small intestine lamina propria | MM | <http://www.ncbi.nlm.nih.gov/pubmed/25621825> |

**List of the U-BIOPRED Consortium project team members**

Uruj Hoda & Christos Rossios, Airways Disease, National Heart & Lung Institute, Imperial College London, UK & Biomedical Research Unit, Biomedical Research Unit, Royal Brompton & Harefield NHS Trust, London, UK; Elisabeth Bel, Faculty of Medicine, University of Amsterdam, Amsterdam, Netherlands; Navin Rao, Janssen Research and Development, High Wycombe, Buckinghamshire, United Kingdom; David Myles, Respiratory Therapy Area Unit, GlaxoSmithKline, Stockley Park, UK; Chris Compton, Discovery Medicine, GlaxoSmithKline, Stockley Park, UK; Marleen Van Geest, AstraZeneca R&D Molndal, Sweden; Peter Howarth & Graham Roberts, Faculty of Medicine, Southampton University, Southampton, UK and NIHR Southampton Respiratory Biomedical Research Unit, University Hospital Southampton, Southampton, UK; Diane Lefaudeux, European Institute for Systems Biology and Medicine, CNRS-ENS-UCBL, Université de Lyon, France; Bertrand De Meulder, European Institute for Systems Biology and Medicine, CNRS-ENS-UCBL, Université de Lyon, France; Aruna T Bansal, Acclarogen Ltd, St John's Innovation Centre, Cambridge, CB4 0WS, UK; Richard Knowles, Knowles Consulting, Stevenage Bioscience Catalyst, Gunnels Wood Road, Stevenage SG1 2FX, UK; Damijn Erzen, Boehringer Ingelheim Pharma, Germany; Scott Wagers, BioSci Consulting, BioSci Consulting, Maasmechelen, Belgium; Norbert Krug, Immunology, Allergology and Clinical Inhalation, Fraunhofer Institute for Toxicology and Experimental Medicine, Hannover, Germany; Tim Higenbottam, Corporate Clinical Development, Chiesi Pharmaceutics Ltd, Cheadle, UK. Current address: Allergy Therapeutics, West Sussex, UK; John Matthews, Genentech Inc, 1 DNA Drive, South San Francisco, CA 94080-4990, USA; Veit Erpenbeek, Translational Medicine - Respiratory Profiling, Novartis Institutes for BioMedical Research, Basel, Switzerland; Leon Carayannopoulos, Merck Inc. Kenilworth, New Jersey, USA; Amanda Roberts, UBIOPRED Patient Input Platform, ELF, Sheffield, UK; David Supple, UBIOPRED Patient Input Platform, ELF, Sheffield, UK; Pim deBoer, UBIOPRED Patient Input Platform, ELF, Sheffield, UK; Massimo Caruso, Department of Clinical and Experimental Medicine Hospital University, University of Catania, Italy; Pascal Chanez, Département des Maladies Respiratoires, Laboratoire d'immunologie, Aix Marseille Université Marseille, France; Sven-Erik Dahlen, The Centre for Allergy Research, The Institute of Environmental Medicine, Karolinska Institute, Stockholm, Sweden; Ildikó Horváth, Department of Pulmonology, Semmelweis University, Budapest, Hungary; Nobert Krug, Fraunhofer Institute for Toxicology and Experimental Medicine Hannover, Germany; Jacek Musial, Dept. of Medicine, Jagiellonian University Medical College, Krakow, Poland; Thomas Sandström, Dept of Medicine, Respiratory and Allergy unit, University Hospital, SE 901 85 Umeå, Sweden.
